# The Ketogenic Diet Improves Gut–Brain Axis in a Rat Model of Irritable Bowel Syndrome: Impact on 5-HT and BDNF Systems

**DOI:** 10.3390/ijms23031098

**Published:** 2022-01-20

**Authors:** Antonella Orlando, Guglielmina Chimienti, Maria Notarnicola, Francesco Russo

**Affiliations:** 1 Laboratory of Nutritional Pathophysiology, National Institute of Gastroenterology “S. de Bellis”, IRCCS Research Hospital, 70013 Castellana Grotte, Italy; antonella.orlando@irccsdebellis.it; 2 Department of Biosciences, Biotechnologies and Biopharmaceutics, University of Bari Aldo Moro, Via Orabona 4, 70125 Bari, Italy; guglielminaalessandra.chimienti@uniba.it; 3 Laboratory of Nutritional Biochemistry, National Institute of Gastroenterology “S. de Bellis”, IRCCS Research Hospital, 70013 Castellana Grotte, Italy; maria.notarnicola@irccsdebellis.it

**Keywords:** 5-HT, animal model, BDNF, irritable bowel syndrome, ketogenic diet

## Abstract

Altered gut–brain communication can contribute to intestinal dysfunctions in the intestinal bowel syndrome. The neuroprotective high-fat, adequate-protein, low-carbohydrate ketogenic diet (KD) modulates the levels of different neurotransmitters and neurotrophins. The aim was to evaluate the effects of KD on levels of 5-HT, the receptors 5-HT_3B_ and 5-HT_4_, the 5-HT transporter SERT, the neurotrophin BDNF, and its receptor TrkB in the colon and brain of a rat model of irritable bowel syndrome (IBS). Samples from Wistar rats exposed to maternal deprivation as newborns and then fed with a standard diet (IBS-Std) or KD (IBS-KD) for ten weeks were analyzed. As controls, unexposed rats (Ctrl-Std and Ctrl-KD) were studied. IBS-Std rats had a disordered enteric serotoninergic signaling shown by increased mucosal 5-HT content and reduced SERT, 5-HT_3B_, and 5-HT_4_ levels compared to controls. In the brain, these animals showed up-regulation of the BDNF receptor TrkB as a counteracting response to the stress-induced reduction of the neurotrophin. KD showed a dual effect in improving the altered 5-HT and BDNF systems. It down-regulated the increased mucosal 5-HT without affecting transporter and receptor levels. KD improved brain BDNF levels and established negative feedback, leading to a compensatory downregulation of TrkB to maintain a physiological steady state.

## 1. Introduction

Irritable Bowel Syndrome (IBS) is a gastrointestinal (GI) functional disorder characterized by GI motor and sensory dysfunctions in the absence of structural abnormalities; it is highly prevalent, chronic, recurrent, and remitting in the population worldwide. Mainly for clinical purposes, IBS is categorized into four different subtypes according to the bowel habits of patients: IBS-D (diarrhea prevalent), IBS-C (constipation prevalent), IBS-M (mixed form), and IBS-U (unspecified) [1].

Chronic pain, anxiety, and depression frequently overlap with IBS [2]. The gut–brain axis is involved in the perception and regulation of visceral sensitivity, linking peripheral GI functions with the sensitive and cognitive centers in the brain [3]. Therefore, altered communication between the two systems can contribute to intestinal dysfunctions (e.g., the induction of visceral pain, disturbed GI motility, the onset of leaky gut, and deregulated entero-endocrine signaling) [4].

As signaling transducers, the neurotransmitter serotonin (5-HT) and the neurotrophin brain-derived neurotrophic factor (BDNF) have been proved to expand their functions outside the central nervous system (CNS) and mediate the signaling with the intestine [5,6].

In the CNS, 5-HT controls mood, sleep, and appetite, whereas it regulates motility, secretion, sensation, inflammation, and the barrier function in the gut. Tryptophan hydroxylases TpH1 and TpH2 synthesize 5-HT in the enterochromaffin cells (ECs) in the intestine and neurons, respectively, composing about 95% of the neurotransmitters concentrated in the gut [7]. The serotonergic signaling relies on several selective receptor subtypes and is terminated via the 5-HT reuptake transporter (SERT) [8].

The pathophysiological role of 5-HT and SERT, together with 5-HT_3_ and 5-HT_4_ receptors, has been widely highlighted in IBS. These two receptors mediate serotonin-induced signaling in the gut [9] and are also expressed in the brain [10]. The mucosal release of 5-HT stimulates internal sensory neurons (most likely through 5-HT_4_ receptors) and external sensory neurons (through 5-HT_3_ receptors) [11]. Previous studies have shown that both 5-HT_3_ and 5-HT_4_ receptor agonists can stimulate GI motility [12]. Like the 5-HT_3_ receptor, the 5-HT_4_ receptor seems to exert multifaceted roles, mainly mediating the relaxation and contraction of circular smooth muscle [13,14].

These molecules have become attractive targets for pharmacological treatments to reduce or promote gut motility, specifically for the IBS-D and IBS-C subtypes [15]. Furthermore, diets fortified with natural compounds (such as berberine or resveratrol) have been recently proposed as a therapeutic intervention to reduce serotonin signaling in IBS patients [16,17,18] and animal models [3].

The neurotrophin BDNF is involved in the development and regeneration of several neuronal populations in the CNS [19], so decreased neuroplasticity can be associated with the psychological comorbidities related to IBS [20]. BDNF is also an essential neurotrophic factor in the GI tract, where it is synthesized by several cell types, such as enterocytes, neurons, and glial cells [21]. Beyond its neuroprotective and neuroplastic roles, it controls visceral sensation, motility, and intestinal barrier functions [22,23]; thus, along with its cognate tropomyosin-related kinase receptor B (TrkB), it plays a role in hypersensitivity conditions via facilitating sensory nerve growth [24] and its interaction with enteroglial cells [25], although with sex-linked differences [26]. Some studies have been performed in rat models of IBS, showing the effectiveness of the BDNF blockage signaling as a possible therapeutic option for visceral hypersensitivity [27,28].

Overall, several findings strongly support the potential beneficial role of a nutritional approach to IBS management [29]. In this context, different data suggest that the high-fat, low-carbohydrate, and adequate-protein ketogenic diet (KD) is not only neuroprotective [30], probably through its effects on mitochondria function, cellular energetics, inflammation, and pain thresholds [31], but it also can modulate the circulating levels of intestinal microbiota and neurotransmitters [32].

On this basis, the present study aimed to evaluate the effects of KD on the levels of 5-HT and BDNF systems in Wistar rats exposed to maternal deprivation (MD) as newborn, as early life stress can induce IBS in adulthood [33].

Considering the importance of the gut–brain interconnections, we evaluated the levels of 5-HT, of the receptors 5-HT_3B_ and 5-HT_4_ and the 5-HT transporter SERT, of the neurotrophin BDNF and its cognate receptor TrkB in colon and brain samples of MD exposed rats fed with a standard diet (IBS-Std) or KD (IBS-KD) for ten weeks. In addition, as control groups, unexposed rats fed with a standard diet (Ctrl-Std) or KD (Ctrl-KD) were also studied.

## 2. Results

### 2.1. Serotonin Levels

**Figure 1:**
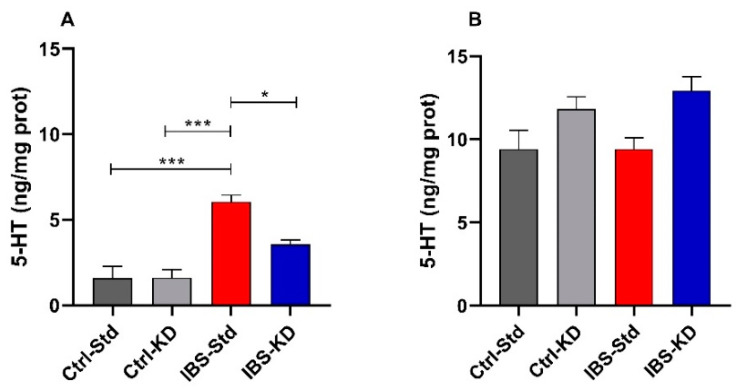
The 5-HT levels are expressed as ng/mg protein in the colon (Panel **A**) and brain (Panel **B**) samples of Ctrl-Std, Ctrl-KD, IBS-Std, and IBS-KD rats, with each group consisting of four rats. Data were analyzed by Kruskal–Wallis analysis of variance and Dunn’s Multiple Comparison Test (* *p* < 0.05; *** *p* < 0.001).

Serotonin levels were determined by ELISA in colon and brain tissue samples from rats belonging to the four experimental groups. In colon samples, the levels of 5-HT were significantly (*p* = 0.0001) 3.8-fold higher in IBS-Std rats (6.06 ± 0.38 ng/mg prot.) compared to both Ctrl-Std (1.58 ± 0.68 ng/mg prot.) and Ctrl-KD ones (1.62 ± 0.47 ng/mg prot.). In the group of IBS rats given KD, the diet appeared to have a neutralizing action as serotonin levels were 1.7 times lower than in the IBS-Std group (*p* = 0.0134; 3.56 ± 0.26 ng/mg prot. vs. 6.06 ± 0.38 ng/mg prot.) (Figure 1, panel A). Conversely, no significant differences in 5-HT levels were found in brain samples among the four groups (Figure 1, panel B).

### 2.2. 5-HT System

The serotonin system was evaluated by Western blot analysis by determining the content of 5-HT_3B_, 5-HT_4_, and SERT proteins expressed as relative intensity (mean ± SEM) of the immunoreactive band in colon and brain tissue samples of Ctrl-Std, Ctrl-KD, IBS-Std, and IBS-KD rats.

**Figure 2:**
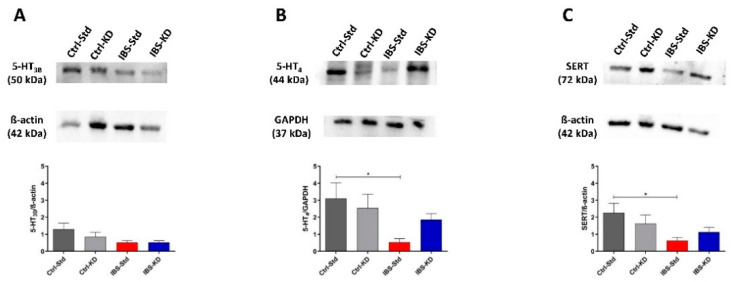
Western blot analysis of 5-HT_3B_ (Panel **A**), 5-HT_4_ (Panel **B**), and SERT (Panel **C**) proteins in colon samples of Ctrl-Std, Ctrl-KD, IBS-Std, and IBS-KD rats, with each group consisting of four rats. Data were analyzed by Kruskal–Wallis analysis of variance and Dunn’s Multiple Comparison Test (* *p* < 0.05).

As for 5-HT_3B_ in the colon, no significant differences were observed among the groups of rats (Figure 2, panel A). On the contrary, 5-HT_4_ protein showed a significant (*p* = 0.0478) six-fold decrease in IBS-Std rats compared to Ctrl-Std ones (0.53 ± 0.21 vs. 3.11 ± 0.91) (Figure 2, panel B). As for the levels of SERT protein, the IBS-Std group showed significantly (*p* = 0.049) reduced (3.6-fold) in comparison with Ctrl-Std rats (0.63 ± 0.20 vs. 2.27 ± 0.56) (Figure 2, panel C).

**Figure 3:**
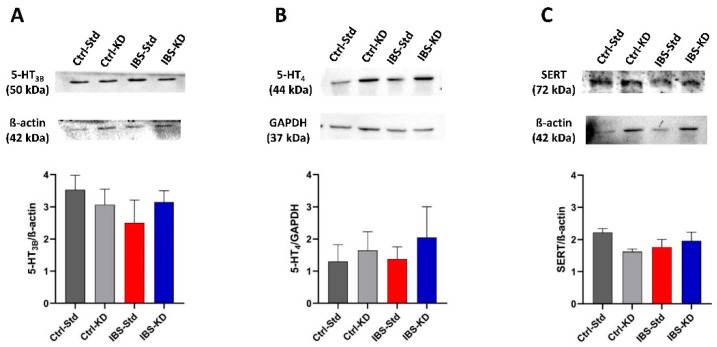
Western blot analysis of 5-HT_3B_ (Panel **A**), 5-HT_4_ (Panel **B**), and SERT (Panel **C**) proteins in brain samples of Ctrl-Std, Ctrl-KD, IBS-Std, and IBS-KD rats, with each group consisting of four rats. Data were analyzed by Kruskal–Wallis analysis of variance and Dunn’s Multiple Comparison Test.

As reported in Figure 3, the content of 5-HT_3B_, 5-HT_4_, and SERT proteins in brain samples of Ctrl-Std, Ctrl-KD, IBS-Std, and IBS-KD rats were not significantly different among the four experimental groups of rats (Figure 3, panel A–C).

### 2.3. BDNF System

The BDNF and TrkB protein levels were evaluated by Western blot analysis in colon and brain samples from the four experimental rat groups and expressed as the immunoreactive bands’ relative intensity (mean ± SEM). In both the tissues, the main BDNF immunoreactive product was a 28 kDa band, corresponding to the stable homodimer formed by the mBDNF form.

**Figure 4:**
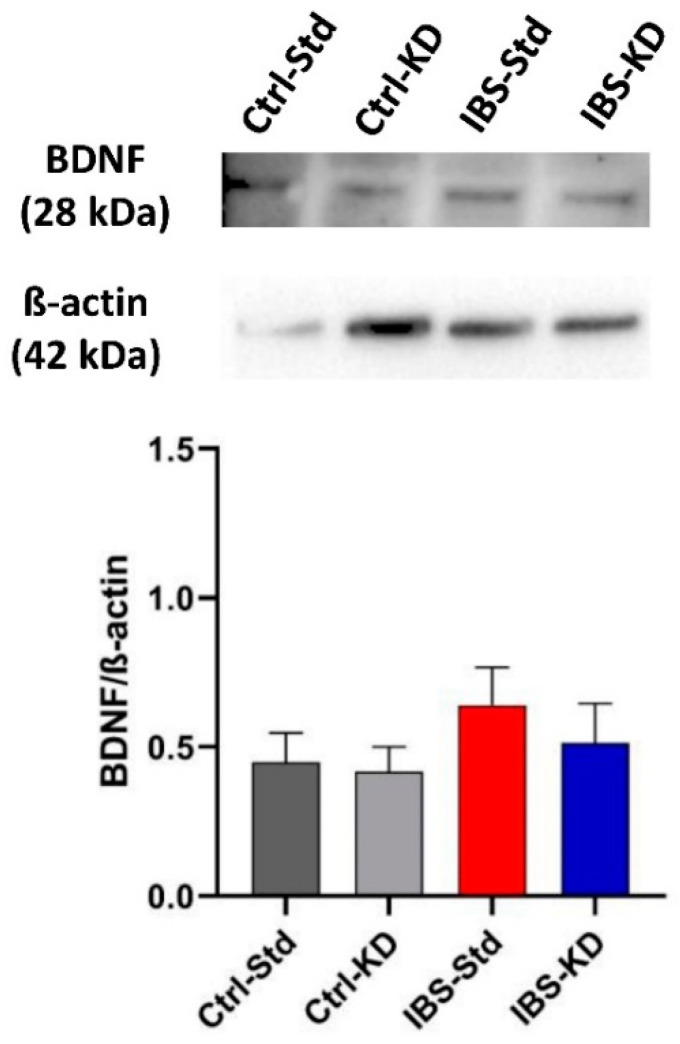
Western blot analysis of BDNF protein in colon samples of Ctrl-Std, Ctrl-KD, IBS-Std, and IBS-KD rats, with each group consisting of four rats. Data were analyzed by Kruskal–Wallis analysis of variance and Dunn’s Multiple Comparison Test.

No significant differences were found in BDNF dimer levels among rat colon samples (Figure 4). Western blot analysis could not highlight any TrkB immunoreactive band in colon samples (data not shown).

**Figure 5:**
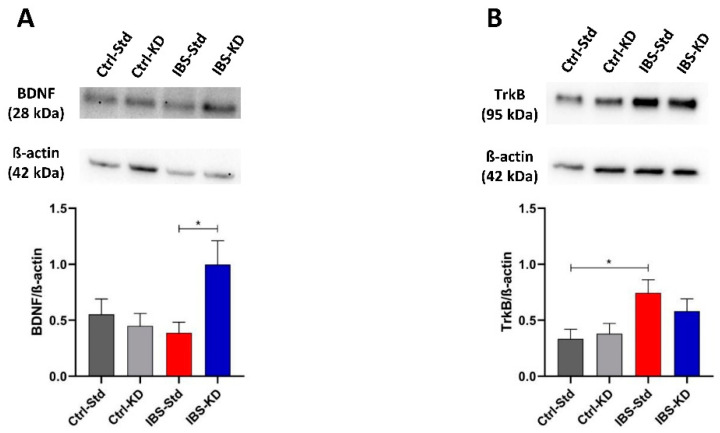
Western blot analysis of BDNF (Panel **A**) and TrkB (Panel **B**) proteins in brain samples of Ctrl-Std, Ctrl-KD, IBS-Std, and IBS-KD rats, each group consisting of four rats. Data were analyzed by Kruskal–Wallis analysis of variance and Dunn’s Multiple Comparison Test (* *p* < 0.05).

In the brain, the levels of the BDNF dimer were different among the four groups of rats, with the minimum value shown by IBS rats fed a standard diet (Figure 5, panel A). The post-test showed the significance of the difference for comparing IBS rats, with the IBS-KD showing a 2.6-fold increased value compared to IBS-Std (*p* = 0.0354; 0.39 ± 0.10 vs. 1.00 ± 0.21). The effects of MD and feeding with KD on the BDNF cognate TrkB receptor levels were evaluated (Figure 5, panel B). A difference was observed in the levels of the TrkB immunoreactive band among the groups, with the statistical significance between Ctrl-Std and IBS-Std, the latter showing a 2.2-fold increased value (*p* = 0.0417; 0.33 ± 0.09 vs. 0.74 ± 0.12).

## 3. Discussion

Recent evidence suggests that changing IBS patients’ eating habits could be proposed as a sustainable and lasting optional approach to drug treatment to ameliorate their symptom profile [29]. Additionally, it is now accepted how altered communication between the gut and the brain can contribute to intestinal and psychological dysfunctions observed in IBS [4]. In this framework, and derived mainly from preliminary results in animal studies [34,35,36], KD could show some interesting therapeutic potentiality.

The animal model used in this research was based on MD in the newborn Wistar rats as a trigger for mimicking IBS in adulthood and the administration of KD characterized by a low-carbohydrate content and high-fat levels. This kind of diet induces a metabolic shift to the use of ketones as a source of energy for cells. It is also depicted as a carbohydrate-restricted diet with a 4:1 ratio of fats to proteins and carbohydrates [37,38].

On this basis, the present study aimed to evaluate whether feeding with KD could counteract the derangement of the gut–brain interconnections in IBS. For this purpose, protein levels of 5-HT, 5-HT3B, and 5-HT4 receptors, the transporter SERT, of the neurotrophin BDNF and its receptor TrkB were evaluated in colon and brain samples of rats that had been exposed to MD for two weeks after birth and then fed with KD for a further ten weeks [29,33].

Results from the present study suggested that two weeks of MD induced abnormalities in the gut–brain axis in adulthood, particularly alterations of 5-HT and BDNF systems in the colon and the brain of the exposed rats. The induced dysfunctions were of different degrees in the two tissues. As reported by Yu et al. [3], a combination of several chronic and acute stressors leads to significant alterations of 5-HT-dependent signaling in both the periphery and in the CNS of the exposed rats. The design of the present study comprised a less severe treatment, consisting only of maternal deprivation. However, the observed changes reached statistical significance depending on the organ where the molecules are most represented or mainly exert their functions since 5-HT significantly changed only in the gut where it is wholly localized [7], while BDNF changed in the brain. In fact, BDNF, although abundant in the periphery [21], exerts its primary function as a neurotrophin on neuronal populations in the CNS [19].

Overall, these results indicate a relationship between the extent of stress and the induced abnormalities of the gut–brain interconnections, and it is interesting to note that the dysfunctions were opposite in their consequences. In the colon of IBS-Std rats, 5-HT appeared to increase relative to controls, while BDNF decreased in the brain. The significantly increased mucosal 5-HT content in IBS-Std rats was associated with reduced SERT, 5-HT_3B_, and 5-HT_4_ receptors compared to controls. This evidence well fits with the hypothesis formulated by Camilleri et al. [39] to explain the disordered enteric serotoninergic signaling in IBS, since it appears that a failure to inactivate the increased serotonin leads to a down-regulation of the receptors.

As concerns the neurotrophin, no firm conclusion can be drawn from the literature regarding the levels of the BDNF protein both in the colon of IBS patients and in animal models. Immunohistochemical data suggested increased BDNF mucosal levels in IBS patients [24], whereas Western blot analysis did not show significant differences between patients and controls [26]. From our data on rats, only a slight increase in BDNF protein in the colon could be inferred in the setting of MD-induced IBS. Other authors have demonstrated through Western blot analysis the significantly increased colon BDNF, however, in a rat model of IBS receiving chronic acute combining stress [3] and in a mouse model of IBD [40]. In our colon samples, we could not identify a TrkB immunoreactive band, agreeing with the reported deficient expression levels of the protein in the colon [41].

The present study showed a concerted response to MD of BDNF and its receptor in the brain. In IBS rats fed a standard diet, levels of the neurotrophin were the lowest, whereas those of TrkB were the highest compared to the other experimental groups. It can be assumed that an up-regulation of the receptor was a counteracting response to the stress-induced reduction of brain BDNF, as already demonstrated in another model of a stressed animal [42].

The KD-associated reduction in carbohydrates forces the body to primarily use fat as fuel by miming the fasting state. It is used as a dietary intervention to treat several neurological and psychiatric disorders [30], although its role in treating other diseases such as nonalcoholic fatty liver disease [43], or even as an adjuvant treatment for cancer, is now emerging [44]. The beneficial effects of the intervention appear to rely on, among the other proposed mechanisms [31,32], the ability of KD to decrease biogenic amines such as 5-HT [45]. Furthermore, it has been shown that circulating levels of BDNF are influenced by calorie restriction in obese subjects [46], and more specifically by dietary PUFA, with a significant role also played by genetics, as shown in celiac patients [47]. Additionally, circulating and brain BDNF levels have been shown to increase with β-oxidation of free fatty acids, along with the short-chain fatty acid β-hydroxybutyrate that is produced in the setting of carbohydrate restriction, fasting, or hypoglycemia, at least in subjects attempting prolonged and exhaustive physical exercise [48,49]. Data concerning the effect of dietary fat on the levels of BDNF in the gut are scarce; it has only recently been demonstrated that a high-fat diet induces a reduction of the expression of BDNF in duodenal neurons [50].

As recently published elsewhere by our group, we have already demonstrated the effectiveness of KD in ameliorating intestinal membrane functions and gut mitochondrial biogenesis in the animal model of IBS, along with the inflammatory status [35,36].

This study showed a dual effect of KD in improving the altered 5-HT and BDNF systems. Dietary intervention was able to down-regulate the increased 5-HT observed in the colon of IBS-Std, although without reaching the levels of controls. KD feeding did not affect the 5-HT transporter and receptors levels.

The feeding with KD improved brain BDNF levels in IBS rats and established negative feedback, leading to a compensatory downregulation of TrkB to maintain a physiological steady state. In addition, the present study found that KD does not significantly affect serotonin and BDNF systems in control rats, neither in colon nor in brain samples. Indeed, a positive role of ketosis in enhancing performance and cognition in the absence of underlying disorders appears to rely only on anecdotal evidence. A recent study showed that nutritional ketosis does not affect healthy individuals’ cognitive function, sleep, or mood [51]. Furthermore, Brownlow et al. [52] have demonstrated that KD could affect metabolic parameters associated with energy metabolism but not hippocampal BDNF in control rats not subjected to stress.

The present paper offers an interesting picture in the colon and brain of the effect of early stress on some molecules involved in the communications between the two districts and the possible curative effect of the KD. Other than the fact that the state of ketosis and the ketonemia levels, which is very important especially in the management of pathologies with a neurological component, were not evaluated, another major drawback of this study is that possible variations have been investigated only at protein levels. So, the results reported herein are mandatory for further studies aiming to deeply analyze the complex pattern of the regulation of 5-HT and BDNF systems in order to shed light on the mechanisms leading to the stressed phenotype and the role of the dietary intervention. Although the current data were obtained in an animal model, which it can mimic the symptoms of IBS and therefore is useful for better identifying the molecular mechanisms of etiopathogenesis and potentially beneficial treatments [33], this does not faithfully reproduce what happens in humans. Undoubtedly, there is need to confirm these results in extensive clinical studies conducted on patients with IBS, also paying attention to the critical role of genetics as a possible modifier [47].

## 4. Materials and Methods

### 4.1. Animals and Experimental Design

The study was approved by the Italian Ministry of Health (approval date: 28 November 2018, n. 901/2018-PR) according to European Union guidelines (Directive 2010/63/E.U. for animal experiments).

The animals (Wistar rats) were housed at the animal facility of the National Institute of Gastroenterology “S. de Bellis” Research Hospital, Castellana Grotte, Bari, Italy. All the applied procedures followed the International Guidelines for using laboratory animals, minimizing animal suffering.

The IBS in adulthood was induced using the animal model of the newborn rats subjected to early-life stress through MD [33]. Postnatal Day 0 (PND 0) was considered as the birthday. Within the PNDs 2 to 14, the puppies experienced MD for 3 h a day.

After weaning, the animals subjected to MD were further divided into two subgroups, one group fed a standard diet (IBS-Std), and one group fed a low-carbohydrate, high-fat ketogenic diet (IBS-KD). The experimental design also included a control group of animals without MD and fed a standard diet (Ctrl-Std) and a group fed a low-carbohydrate, high-fat ketogenic diet (Ctrl-KD) (Table 1). Only male rats were considered for this study.

Diets (4RF21 standard diet and ketogenic diet) were purchased by Mucedola Srl, Settimo Milanese, Italy, and administered ad libitum for ten weeks after PND 14. Their composition is shown in Table 2.

Rats were checked every day, evaluating different parameters regarding the degree of suffering and stress induced experimentally (blepharospasm, hollow cheeks, abnormal position of the ears and the whiskers, appetite loss, and liquid stools). Each parameter was recorded, attributing a score from 0 (absent) to 2 (evident) to calculate the possible onset of pain and suffering.

All animals in the study did not show any of the above stress-related symptoms, except for a slowdown in the growth of puppies with MD, which had lower weights than the control-group puppies.

After treatment, the animals were sacrificed by inhalation of an overdose of isoflurane, and both colon and brain samples were immediately removed and stored at −80 °C until assayed.

### 4.2. Serotonin Levels

The serotonin levels in colon and brain samples from all the experimental groups of rats were evaluated using the enzyme-linked immunosorbent assay (Elisa) kits (MyBioSource, San Diego, CA, USA), following the manufacturer’s instructions.

### 4.3. Western Immunoblotting

Protein extracts were obtained using a standard procedure from colon and brain samples of Ctrl-Std, KD, IBS-Std, and IBS-KD rats. For Western blot analysis, the aliquots of 50 µg of total protein extracts from each sample were loaded into 10% pre-cast polyacrylamide gels (Bio-Rad, Milan, Italy). Anti-5-HT_3B_, anti-5-HT_4_, anti-SERT (Abcam, Cambridge, UK), anti-BDNF (MyBioSource, San Diego, CA, USA), anti-TrkB (Thermo Scientific, Rockford, IL, USA), anti-β-actin, and anti-GAPDH (Cell Signaling, Danvers, MA, USA) were used as primary antibodies. The proteins were detected by chemiluminescence (ECL, Bio-Rad, Milan, Italy), and the densitometric analysis of each protein-related signal was obtained using the Molecular Imager Chemidoc^TM^ (Bio-Rad, Milan, Italy) and normalized against β-actin or GAPDH expression.

### 4.4. Statistical Analysis

Due to the non-normal distribution of the data, nonparametric tests were performed. Data were analyzed by Kruskal–Wallis analysis of variance and Dunn’s Multiple Comparison Test. Differences were considered significant at *p* < 0.05. All data represent the results of at least two independent experiments and are expressed as mean ± SEM. A specific statistical package was used for the exact nonparametric inference (StataCorp 2005; Stata Statistical Software: Release 9, College Station, TX, USA).

## 5. Conclusions

In conclusion, although partial, the results from this paper provide preliminary evidence of the efficacy of KD in improving imbalanced gut–brain interconnections, thereby reducing the damaging effects of stress in an animal model of IBS. This finding could have future clinical repercussions since the possibility of interrupting the dysfunction of the brain-gut axis through specific dietary treatments could allow new therapeutic strategies based on their neuroprotective properties.

## Figures and Tables

**Table 1:** Experimental groups: no irritable bowel syndrome (IBS) rats fed a standard diet (Ctrl-Std); no IBS rats fed a low-carbohydrate, high-fat ketogenic diet (Ctrl-KD); IBS rats fed a standard diet (IBS-Std); IBS rats fed a low-carbohydrate, high-fat ketogenic diet (IBS-KD). Maternal deprivation (3 hours/day from Postnatal Day (PND) 2 to 14). Treatment (for ten weeks after PND 14).

Group	Rats (Number)	Maternal Deprivation (3 h/Day from PNDs 2 to 14)	Treatment (for Ten Weeks after PND 14)
Ctrl-Std	12	No	Standard diet
Ctrl-KD	13	No	Ketogenic diet
IBS-Std	11	Yes	Standard diet
IBS-KD	17	Yes	Ketogenic diet

**Table 2:** Main constituents of Standard Diet and Ketogenic Diet.

Analytical Constituents	Standard Diet	Ketogenic Diet
Moisture	12%	0%
Crude protein	18.5%	16.0%
Crude oils and fats	3.0%	67.0%
Crude fibers	6.0%	6.0%
Crude ash	7.0%	4.5%

## Data Availability

The data that support the findings of this study are available from the corresponding author upon reasonable request.
